# Editorial: The Uncultured Microorganisms: Novel Technologies and Applications

**DOI:** 10.3389/fmicb.2021.756287

**Published:** 2021-11-24

**Authors:** Bin-Bin Xie, Meng Li, Karthik Anantharaman, Nikolai V. Ravin

**Affiliations:** ^1^Microbial Technology Institute and State Key Laboratory of Microbial Technology, Shandong University, Qingdao, China; ^2^Archaeal Biology Center, Institute for Advanced Study, Shenzhen University, Shenzhen, China; ^3^Department of Bacteriology, University of Wisconsin-Madison, Madison, WI, United States; ^4^Institute of Bioengineering, Research Center of Biotechnology of the Russian Academy of Sciences, Moscow, Russia

**Keywords:** uncultured microorganisms, cultivation techniques, single-cell genomics, metagenomics, microbial diversity

Although our knowledge of microbial diversity has been greatly advanced in recent decades, the majority of microorganisms remain uncultured and therefore poorly characterized. Two major approaches have been used in the study of uncultured microorganisms: cultivation-dependent and cultivation-independent. The later approach is based on the analysis of genomes assembled from metagenomes or obtained from single cells, providing predictions regarding the organism's metabolic capabilities and ecological functions (Rinke et al., 2013; Woyke et al., 2017; Nayfach et al., 2021). Application of cultivation-independent approaches to natural environments has greatly expanded the tree of life and revealed novel microbial lineages, most of which still has no cultured members (Rinke et al., 2013; Castelle and Banfield, 2018). However, comprehensive understanding of the biology of these microorganisms and their functions in environment requires the pure cultures (or co-cultures) and thus development of novel cultivation technologies (Sun et al., 2020; Lewis et al., 2021). With both approaches, rapid progress has been made in the field due to the development and application of a variety of new technologies, including *in situ* cultivation, high-throughput cultivation, application of resuscitation stimuli, cultivation after physical cell sorting, metagenomics and other “omics” technologies (Salam et al., 2021). Moreover, information that can be extracted from multi-omics data can be applied for the targeted isolation of yet uncultured microorganisms (Gutleben et al., 2018).

This Research Topic deals with recent advances in the cultivation, diversity, and physiology of previously uncultured microorganisms, with emphasis on developments and applications of novel technologies. It consists of six works.

The dilution-to-extinction culturing approach has been used in the cultivation of a number of oligotrophic marine bacteria since its early success in cultivating the marine SAR11 clade bacteria (Rappe et al., 2002). This approach was used to cultivate freshwater bacteria at a large scale in the work of Kim et al. In their work, bacterial growth was recorded for 14% of the total 5,376 inoculated wells. Using 16S rRNA gene sequencing, they showed that a large number of isolates belong to previously uncultured or underrepresented freshwater bacterial groups, including the acI, acIV, LD28, FukuN57, MNG9, and TRA3-20 lineages.

Some bacteria can produce molecules that promote or stimulate the growth of other bacteria. One such molecule called the resuscitation-promoting factor (Rpf) can induce bacterial resuscitation. In this Topic, Lopez Marin et al. showed that the heat-liable component of the *Micrococcus luteus* culture supernatant (containing Rpf) can increase the number and diversity of cultured bacterial taxa from a soil sample. This work also showed that the supernatant treatment allowed the cultivation of 51 previously uncultured potentially novel bacterial species.

The reasons for failing to cultivate microorganisms include the lack of correct nutrients, insufficient incubation time, inappropriate temperature or pH, need of specific growth signals, dependence on other microorganisms and many other factors. In their paper, Dahal et al. reported isolation of a bacterium, designated strain G-1-1-14^T^, from forest soil using low nutrient medium and long enrichment time. This strain represents a novel species for which the name *Azohydromonas caseinilytica* sp. nov. was proposed.

As a cultivation-independent method, 16S rRNA gene amplicon sequencing is the major approach in the elucidation of microbial diversity and the community structure at present. In the work of Wu et al., 16S rRNA gene amplicon sequencing was used to determine the composition of the gut microbiota of captive and wild oriental white storks. Their work revealed differences in the composition and diversity of gut microbiota under different breeding conditions.

With advances in sequencing technologies and bioinformatics approaches, hundreds and thousands of microbial genomes can be obtained from environmental metagenomes (Nayfach et al., 2021). Zhou et al. produced approximately 145 gigabases of metagenomic sequence data for the fecal samples from 11 Tibetan pigs, an important domestic animal in the Qinghai-Tibet Plateau. *De novo* assembly and binning recovered 322 metagenome-assembled genomes assigned to 11 bacterial and 2 archaeal phyla. 191 of these genomes represented the uncultivated microorganisms derived from novel prokaryotic taxa. Over 13,000 genes for carbohydrate-active enzymes were identified providing an expanded repertoire of biomass-degrading genes for future biotechnological applications.

Soil microorganisms historically have been a rich resource for natural product discovery, yet our knowledge of their biosynthetic gene clusters (BGCs) has been limited by culture- and PCR-biases. Santana-Pereira et al. applied so-called functional metagenomic approach to identify the biosynthetic potential of soil microorganisms. They constructed a large-insert metagenomic library in *Escherichia coli* from soil and screened the library clones for biosynthetic gene clusters (BGCs) using either PCR or a next generation sequencing. A total of 1,015 BGCs were detected from 19,200 clones, identifying 223 clones that carry a polyketide synthase and/or non-ribosomal peptide synthetase clusters, a dramatically improved hit rate compared to PCR screening. This study provided novel resources for natural product discovery and revealed heretofore undiscovered functional and phylogenetic diversity that have been captured in soil genomic resources.

## Author Contributions

B-BX, ML, KA, and NR co-edited the Research Topic and wrote the editorial. All authors approved the submitted version.

## Funding

B-BX was funded by the Young Scholars Program of Shandong University (Grant No. 2016WLJH41) and the Youth Interdisciplinary Science and Innovative Research Groups of Shandong University (Grant No. 2020QNQT006). ML was funded by the Innovation Team Project of Universities in Guangdong Province (Grant No. 2020KCXTD023) and the Shenzhen Science and Technology Program (Grant No. JCYJ20200109105010363). NR was funded by the Russian Science Foundation (Grant No. 19-14-00245).

## Conflict of Interest

The authors declare that the research was conducted in the absence of any commercial or financial relationships that could be construed as a potential conflict of interest.

## Publisher's Note

All claims expressed in this article are solely those of the authors and do not necessarily represent those of their affiliated organizations, or those of the publisher, the editors and the reviewers. Any product that may be evaluated in this article, or claim that may be made by its manufacturer, is not guaranteed or endorsed by the publisher.
